# Towards an Ethical Framework for Publishing Twitter Data in Social Research: Taking into Account Users’ Views, Online Context and Algorithmic Estimation

**DOI:** 10.1177/0038038517708140

**Published:** 2017-05-26

**Authors:** Matthew L Williams, Pete Burnap, Luke Sloan

**Affiliations:** Cardiff University, UK; Cardiff University, UK; Cardiff University, UK

**Keywords:** algorithms, computational social science, context collapse, ethics, social data science, social media, Twitter

## Abstract

New and emerging forms of data, including posts harvested from social media sites such as Twitter, have become part of the sociologist’s data diet. In particular, some researchers see an advantage in the perceived ‘public’ nature of Twitter posts, representing them in publications without seeking informed consent. While such practice may not be at odds with Twitter’s terms of service, we argue there is a need to interpret these through the lens of social science research methods that imply a more reflexive ethical approach than provided in ‘legal’ accounts of the permissible use of these data in research publications. To challenge some existing practice in Twitter-based research, this article brings to the fore: (1) views of Twitter users through analysis of online survey data; (2) the effect of context collapse and online disinhibition on the behaviours of users; and (3) the publication of identifiable sensitive classifications derived from algorithms.

## Introduction

The recent surge in social media uptake and the programmatic availability of vast amounts of ‘public’ online interactional data to researchers have created fundamental methodological and technical challenges and opportunities for social science. These challenges have been discussed methodologically, conceptually and technically (see Burnap et al., 2015b; Edwards et al., 2013; Ruppert et al., 2013; Savage and Burrows, 2009; Sloan et al., 2013; Williams et al., 2013, 2017). However, there is an additional dimension that has received limited engagement in the sociology literature: the challenge of ethics (see Beninger et al., 2014; Metcalf and Crawford, 2016; Townsend and Wallace, 2016; Williams, 2015). The emerging consensus is that the digital revolution has outpaced parallel developments in research governance and agreed good practice. Codes of ethical conduct that were written in the mid-20th century are being relied upon to guide the collection, analysis and representation of digital data in the 21st century. While these codes have been informed by recent writings on some forms of Internet research (see Buchanan and Zimmer, 2016 and Eynon et al., 2016 for extensive overviews), social media presents new challenges. Researchers across the social sciences are routinely harvesting Twitter data and publishing identifiable content of communications and the computed classifications of algorithms without consent. For example, Awan (2014), Innes et al. (2016) and Roberts et al. (2017) published highly sensitive Twitter content without any valid attempt to protect the privacy or, to the best of our knowledge, to gain the informed consent of users.^1^ These and other papers fail to include a single mention of the ethics of conducting social media research, leaving open the questions whether these researchers had effectively engaged with existing learned society guidelines or the emerging literature in this area. What is deeply problematic about these practices is that they have the potential to make sensitive personal information identifiable beyond the context it was intended for, and under some conditions, the publication of these data may expose users to harm.

Twitter is particularly ethically challenging because of the partial free availability of the data. Terms of service specifically state users’ posts that are public will be made available to third parties, and by accepting these terms users legally consent to this. In this article we argue researchers must interpret and engage with these commercially motivated terms of service through the lens of social science research that implies a more reflexive approach than provided in legal accounts of the permissible use of these data in publications. This article presents an analysis of Twitter users’ perceptions of research conducted in three settings (university, government and commercial), focusing on expectations of informed consent and the provision anonymity in publishing user content. The central arguments of the article are that ethical considerations in social media research must take account of users’ expectations, the effect of context collapse and online disinhibition on the behaviours of users, and the functioning of algorithms in generating potentially sensitive personal information.

## Context

The global adoption of social media over the past half a decade has seen ‘digital publics’ expand to an unprecedented level. Estimates put social media membership at approximately 2.5 billion non-unique users, with Facebook, Google+ and Twitter accounting for over half of these (Williams et al., 2017). These online populations produce hundreds of petabytes^2^ of interactional data daily. Despite early concern that these forms of data were a distraction from more important sociological labour, it is now largely accepted that these interactions constitute a socio-technical assemblage that creates a new public sphere where key aspects of civil society are played out. The impact of the increase in social interaction via the machine interface on sociality has been discussed for some time (Beer, 2009; Lash, 2001, 2007). Lash (2001) intrinsically meshes together life and technology, stating how the latter mediates an increasing amount of interaction, through email, mobile devices and the like. Technology becomes a way of life, a way of doing things. Without this technological interface, individuals cannot access aspects of their life or culture which is now at-a-distance. In this view, culture and technology are intrinsically linked. Urry (2000) writes of a transition from ‘social as society’ to ‘social as mobility’ reflected by flows of people and data in time and space, reshaping relations around objects, as opposed to older ties of class, race, gender and place. Knorr-Cetina (2001) calls this ‘object orientated practice’ which is useful in understanding new forms of social organisation, change and identity in digital society. Tinati et al. (2014) propose that social media in general, and specifically Twitter, offer potential for exploring empirical work that begins to unpack new social relations that are orientated around digital subjects and objects.

### New Forms of Data Generation and Collection

Social media researchers have experimented with data from a range of sources, including Facebook, YouTube, Flickr, Tumblr and Twitter to name a few. Twitter is by far the most studied as it differs from other networks, such as Facebook, in that the data are more accessible to researchers. Twitter has become the primary space for online citizens to publicly express their reaction to events, and hence a source of data for social science research into digital publics. The Twitter streaming Application Programming Interface (API) provides three levels of data access: the free random 1 per cent that delivers ~5M tweets daily, and the random 10 per cent and 100 per cent (chargeable or free to academic researchers upon request). Datasets on social interactions on this scale, speed and ease of access have been hitherto unrealisable in the social sciences, and have led to a flood of conference papers and journal articles, many of which include full text content from Twitter communications without informed consent from users. This is presumably because of the follower model and mode of posting facilitated by Twitter, which may lead to the assumption that ‘these are public data’, and are therefore not entitled to the rigour and scrutiny of ethics panels. Even when these data practices are scrutinised, ethics panels may be convinced by the ‘public data’ argument. This article focuses on one key aspect of the social media ethics process, publishing original content from Twitter posts, and outlines users’ perceptions to address some of the potential harms that may stem from using these data in research outputs without anonymity and informed consent.

## Ethics in Social Media Research

### Learned Society Standards

Several learned societies have developed ‘bolt-on’ ethical guidelines for research in digital settings, including the British Sociological Association (BSA),^3^ the British Psychological Society (BPS), the British Society of Criminology (BSC), the British Educational Research Association (BERA) and the European Society for Opinion and Market Research (ESOMAR). The Association of Internet Researchers (AoIR) was the first to develop a dedicated set of guidelines, now in their second iteration. Broadly, most guidelines adopt the ‘situational ethics’ principle: that each research situation is unique and it is not possible simply to apply a standard template in order to guarantee ethical practice. In relation to informed consent BERA specifically state that social networking and other online activities, present challenges for consideration of consent issues and the participants must be clearly informed that their participation and interactions are being monitored and analysed for research. On anonymity, the guidelines state one way to protect participants is through narrative and creative means, which might require the fictionalising of aspects of the research. In relation to consent ESOMAR state that if it has not been obtained researchers must ensure that they report only depersonalised data from social media sources. In relation to anonymity the guidelines state where consent is not possible their analysis must only be conducted upon depersonalised data and if researchers wish to quote publicly made comments they must make reasonable efforts to either seek permission from the user to quote them or mask the comment. These guidelines are reflected upon further in the discussion section.

### Legal Considerations

Data extracted from the Twitter APIs contain personal information meaning they are subject to relevant data protection legislation, including the UK Data Protection Act (DPA). In cases where informed consent cannot be sought from users (likely to be the majority of cases if thousands of posts are being subject to analysis), a social researcher should establish the fair and lawful basis for collecting personal information. A researcher can accept that the terms of service of social media networks provide adequate provision to cover this aspect of the DPA. However, if the data have been collected using a service that provides additional meta data on users, such as *sensitive* personal characteristics (e.g. ethnicity and sexual orientation) based on algorithms that make estimations, the legal issue of privacy may be compounded. Under the DPA *sensitive* personal information can only be processed under certain conditions (see Schedule 3).^4^ Deriving insights and making conclusions about a person or persons’ views (e.g. hate speech) and publicly releasing this information along with the identifiable content of communications could lead to stigmatisation, or to the extreme extent actual bodily harm, should the offline location of the social media persona be established. Within the EU the General Data Protection Regulation will replace the DPA in 2018. It includes provisions for the erasure of personal data and restrictions on data dissemination to third parties. However, it also imposes limitations on the right to be forgotten, including cases in which data are processed for historical, statistical and scientific purposes. To what extent these proposals will impact upon social media research is unclear.

### Socialising Data and the Role of Algorithms

Critical perspectives on human-technology interactions have focused in part on how data are made, acknowledging their inherent social aspect (Beer, 2009; Burrows, 2009; Couldry et al., 2016; Lash, 2007; Ruppert, 2015) and the role of algorithms in the automated classification of users and behaviours (Van Dijck, 2013). A focus is taken on the way in which people, technologies, practices and actions are involved in how data are shaped, made and captured via a set of obfuscated relations. These data are not therefore ‘naturally occurring’, but are instead manufactured by agents that harbour particular sets of priorities. For example, the functions of social media platforms are routinely changed, largely in an effort to improve customer experience. In turn, users adjust their online behaviours to take advantage of new features (e.g. the removal of the 140 character limit on direct messages on Twitter). Lessig (1999), adopting a digital realist perspective, recognises the disruptive capacity of technology within social systems. This argument is related to recent behavioural research on ‘soft’ or ‘asymmetric’ paternalism (also known as *nudges*) that has begun exploring ways of shaping social behaviours via the use of algorithms (Acquisti, 2009). Algorithms are not only routinely used to shape behaviours, but to also *classify* for the purposes of *enhancing* data on users. Van Dijck (2013) shows how social media giants are using algorithms to estimate personal characteristics and viewpoints (sexual orientation, ethnicity, political affiliation and so on) that are not volunteered by users, in an effort to increase the commercial viability of their data products.^5^ Often users are not aware of these classifications as they are held in separate databases that can be purchased by data consumers, including researchers. Beyond social networks, data harvesting tools, such as the Economic and Social Research Council (ESRC) funded *Cardiff Online Social Media Observatory* (COSMOS) platform (Burnap et al., 2015b) and commercially available software (e.g. Ripjar, Dataminr and Pulsar) use algorithms to classify users and behaviours as data are collected from APIs. While some of these platforms reveal to researchers and users how these algorithms operate in the interest of open science (e.g. COSMOS) others obfuscate the code due to commercial interests. One consequence of this is an inability to evaluate the effectiveness of classifications (e.g. the rate of false positives). These ‘data socialities’ (Ruppert, 2015: 1) have profound consequences for the discussion of issues such as privacy/anonymity, informed consent and data ownership. Therefore, a reorientation is required to a ‘social ethics’ that ‘captures the connectedness and interdependent relations that make up and are made up by Big Data’.

### Public Attitudes

Beninger et al. (2014) found that Internet users differ in how familiar and comfortable they are with the privacy and security settings that are provided by social media networks, and recommended researchers should not assume all users have read and understood terms of service that govern issues such as consent and privacy. Users also expressed concern over their photos, Twitter handles (screen names) and personal and sensitive posts being published in research papers. There was an expectation from users that they be approached for consent if there was intent to publish these kinds of data. Evans et al. (2015) conducted a survey of users’ attitudes towards social media research in government and commercial settings. Three in five respondents reported knowing that their social media data (across all platforms) could be shared with third parties under the terms of service they sign up to. However, near two-thirds felt that social media data should not be shared with third parties for research purposes. These views softened when users were offered anonymity where they were quoted in publication and where only public data (such as public Twitter posts) were to be used in the research. To date no academic research has statistically modelled the predictors of the views of users towards the use of their Twitter posts in various settings.

## Methods and Measures

### Data and Modelling

The primary quantitative data used in this analysis were derived from an online survey of 564 members of the Twitter using public in the UK. The Bristol Online Survey tool^6^ was used to design and distribute the questionnaire via social networks. Nonprobability sampling was employed to derive the sample of respondents. As the hypotheses tested in this analysis are concerned more with the existence of inter-variable relations and strengths of association than estimating population prevalence, the use of nonprobability sampling does not fundamentally weaken the design of the study (Dorofeev and Grant, 2006). Moreover, our study is principally concerned with ‘soft’ measures (attitudes, perceptions, opinions), which have no absolute validity (they cannot be compared with any authoritative external measure). However, sampling bias can still impact analysis if a sample is significantly uncharacteristic of the target population. The sample does not deviate significantly from what we know about the population of Twitter users in the UK. As our sample reflects, Twitter users are more likely to be younger, low to middle income earners, and are less likely to have children as compared to the general population in the UK (Sloan, 2017; Sloan and Morgan, 2015; Sloan et al., 2013, 2015). However, given the size of the sample and the violation of the normality assumption for ordered linear regression analysis, the bias corrected and accelerated bootstrapping technique was utilised.^7^ The authors established informed consent via the introduction page to the online survey. Those under 18 were asked not to complete the survey.

### Dependent Measures

Table 1 details the five measures that were used as dependent variables in the models. First, survey respondents were asked to express their concern on an ordinal scale with regards to their Twitter data being used by researchers in three settings: universities, government departments and commercial organisations.^8^ Second, respondents were asked to express their agreement on an ordinal scale with regards to the statements ‘If any of my Twitter posts are published in academic research I would expect to be asked for my consent’ and ‘If any of my Twitter posts are published without my consent for academic research my identity should be anonymised.’

**Table 1.** Sample descriptives (*N* = 564).

	Coding	%/*M*^a^	*N*^b^/SD
*Dependent variables*			
Concern – university research	Not at all concerned	37.2	136
	Slightly concerned	46.4	170
	Quite concerned	11.2	41
	Very concerned	5.2	19
Concern – government research	Not at all concerned	23.3	85
	Slightly concerned	27.7	101
	Quite concerned	25.5	93
	Very concerned	23.6	86
Concern – commercial research	Not at all concerned	16.8	61
	Slightly concerned	32.1	117
	Quite concerned	29.4	107
	Very concerned	21.7	79
Expect to be asked for consent	Disagree	7.2	26
	Tend to disagree	13.1	47
	Tend to agree	24.7	89
	Agree	55.0	198
Expect to be anonymised	Disagree	5.1	18
	Tend to disagree	4.8	17
	Tend to agree	13.7	48
	Agree	76.4	268
*Independent variables*			
Frequency of posts daily	Scale (range: 1 ‘Less than once’ to 7 ‘over 10’)	1.75	1.23
Post personal activity	Yes = 1	37.7	161
Post personal photos	Yes = 1	19.0	81
Knowledge of terms of service (ToS) consent	Yes = 1	75.5	317
Net use (years)	Scale (range: 1 ‘Less than year’ to 9 ‘15+ years’)	6.59	1.76
Net use (hours per day)	Scale (range: 1 ‘Less than hour’ to 10 ‘10+ hours’)	6.03	2.52
Net skill	Scale (range: 1 ‘Novice’ to 10 ‘Expert’)	7.69	1.60
Sex	Male = 1	48.93	276
Age	Scale (range: 18 to 83)	25.38	10.17
Sexual orientation	Heterosexual = 1	83.6	357
Ethnicity	White = 1	91.1	389
Relationship status	Partnered = 1	45.4	194
Income	Scale (range: 1 ‘below 10K’ to 11 ‘100K+’)	3.72	3.07
Has child under 16	Yes = 1	7.3	31

*Notes*: ^a^Mean and Standard Deviation given for scale variables; ^b^Reduction in sample size due to missing data.

### Independent Measures

Table 1 also details the independent measures entered into the regression models. Respondents were asked to detail their frequency of Twitter posts; type of post (posting textual information on personal activities and posting personal photographs); awareness of Twitter terms of service (including agreement to consent to share information with third parties); Internet usage patterns (number of years using the Internet, number of hours per day using the Internet and self-perceived level of Internet expertise); and demographic characteristics (sex, age, sexual orientation, ethnicity, relationship status, income and whether the respondent has children under 16).

The independent variables were regressed onto the five dependent variables using ordered logistic regression. Results from correlational analyses, and tolerance statistics and variance inflation factors showed there were no problems with multi-collinearity among the predictor variables. Statistics indicated a robust fit to the data in both models (see Tables 2 and 3).

**Table 2.** Ordered regression predicting concern about using Twitter data in three research settings.

	University		Government		Commercial	
	B	Exp(B)	B	Exp(B)	B	Exp(B)
Frequency of posts	0.048	1.05	−0.067	0.94	−0.227	0.80
Post personal activity	0.216	1.24	0.101	1.11	0.470	1.60
Post personal photos	0.132	1.14	0.119	1.13	0.133	1.14
Knowledge of ToS consent	−0.465	0.63	−0.246	0.78	−0.289	0.75
Net use (years)	−0.041	0.96	−0.059	0.94	0.059	1.06
Net use (hours per day)	0.092	1.10	0.089	1.09	0.062	1.06
Net skill	0.078	1.08	0.149	1.16	0.135	1.14
Sex	−0.659	0.52	−0.291	0.75	−0.353	0.70
Age	0.003	1.00	0.072	1.07	0.066	1.07
Sexual orientation	−0.27	0.76	−0.752	0.47	−0.653	0.52
Ethnicity	0.055	1.06	−0.311	0.73	−0.422	0.66
Relationship status	−0.414	0.66	−0.206	0.81	−0.363	0.70
Income	−0.045	0.96	−0.045	0.96	−0.053	0.95
Has child under 16	0.846	2.33	0.27	1.31	0.498	1.65
Model fit						
−2 log likelihood	790.730		944.497		920.106	
Model chi-square	31.182		65.712		66.789	
d.f.	15		15		15	
sig.	0.00		0.00		0.00	
Cox and Snell pseudo R²	0.08		0.17		0.17	
Nagelkerke pseudo R²	0.09		0.18		0.18	

**Table 3.** Ordered regression predicting expectation of request for informed consent and anonymity in Twitter research in university settings.

	Informed consent		Anonymity	
	B	Exp(B)	B	Exp(B)
Frequency of posts	−0.05	0.95	−0.097	0.91
Post personal activity	0.034	1.03	0.311	1.36
Post personal photos	−0.272	0.76	0.471	1.61
Knowledge of ToS consent	−0.478	0.62	0.115	1.12
Net use (years)	−0.155	0.86	−0.105	0.90
Net use (hours per day)	0.055	1.06	0.049	1.05
Net skill	−0.063	0.94	−0.109	0.90
Sex	−0.241	0.79	−0.385	0.68
Age	−0.020	0.98	0.017	1.02
Sexual orientation	−0.167	0.85	0.004	1.00
Ethnicity	0.160	1.17	−1.369	3.90
Relationship status	−0.019	0.98	−0.129	0.88
Income	−0.021	0.98	−0.004	1.00
Has child under 16	0.243	1.27	0.052	1.05
Model fit				
−2 log likelihood	788.767		526.805	
Model chi-square	24.762		18.68	
d.f.	15		15	
sig.	0.00		0.00	
Cox and Snell pseudo R²	0.09		0.09	
Nagelkerke pseudo R²	0.09		0.09	

## Findings

Table 1 provides details of both the dependent and independent variables used in this study. Ninety-four per cent of respondents were aware that Twitter had terms of service, and just below two-thirds had read them in whole or in part. Seventy-six per cent knew that when accepting terms of service they were providing consent for some of their information to be accessed by third parties. Least concern was expressed in relation to Twitter posts being used for research in university settings (84% of respondents were not at all or only slightly concerned, compared to 16% who were quite or very concerned). Concern in relation to Twitter being used for research rose in government (49% were quite or very concerned) and commercial settings (51% were quite or very concerned). Respondents expressed high levels of agreement in relation to the statements on consent and anonymity in Twitter research. Just under 80 per cent of respondents agreed that they would expect to be asked for their consent before their Twitter posts were published in academic outputs. Over 90 per cent of respondents agreed that they would want to remain anonymous in publications stemming from Twitter research based in university settings.

Table 2 details the results from the ordered logistic regression models on the concern measure in three research settings. In relation to university settings, those with no knowledge of Twitter’s terms of service consent clause were more likely to express concern (odds increase of 1.59). Those who use social media to post personal activity and personal photographs, and those who use the Internet for more hours in the day were also more likely to express concern (odds increase of 1.24, 1.14 and 1.10 respectively), but the effects are marginal. Of the demographic variables, parents of children under 16 were more likely to be concerned compared to non-parents (odds increase of 2.33), and female respondents were more likely to be concerned compared to male respondents (odds increase of 1.92), possibly reflecting recent high-profile instances of Twitter ‘trolling’ targeted at women (e.g. the Gamergate controversy and the harassment of Caroline Criado-Perez).

Several variables emerged as exerting a notable effect on concern in government and commercial settings. Lesbian, gay and bisexual (LGB) respondents were more likely to express concern over their Twitter posts being used in government (odds increase of 2.12) and commercial settings (odds increase of 1.92), compared to heterosexual respondents. Again, high-profile cases of Twitter abuse against members of the LGB and transgender communities (including the Olympic diver Tom Daley) may have an impact on the perceptions held by these respondents. However, it is also likely that the historic marred relationship between the LGB community and the state (see Weeks, 2007) may be an overriding factor generating this distrust in government organisations using online data. Older respondents were also more likely to report higher degrees of concern in both these settings, as were those who had a higher level of Internet expertise. Those who posted information most often on Twitter and those who did not post personal messages were less likely to be concerned with their information being used in commercial settings.

Table 3 details the results of the ordered logistic regression models for agreement towards the consent and anonymity statements. Those respondents who reported familiarity with Twitter’s terms of service consent clause were less likely to expect to be asked for their informed consent by university researchers to publish content (odds decrease of 0.62). Early adopters of the Internet were likely to hold the same view, but to a lesser degree. Parent, female and LGB Twitter users were more likely to expect to be asked for their informed consent (odds increase of 1.27, 1.27 and 1.18 respectively). Female Twitter users, and those who post personal messages and photos were more likely to expect anonymity in publishing (odds increase of 1.47, 1.36 and 1.61 respectively). In particular, Black or Minority Ethnic (BME) tweeters were much more likely (odds increase of 3.90) to want anonymity compared to white tweeters. Combined, these findings lend support to the position that informed consent should be obtained ahead of publishing Twitter posts, especially when personal information (e.g. extreme opinion, photos, demographic information, location, etc.) is directly quoted in publication. Quantitative analysis of Twitter data that presents findings in aggregate form (such as tables of regression results, topic clusters in word clouds and anonymised network visualisations) is one way to support ethical research without the need for informed consent. However, ethics are compounded in qualitative Twitter research due to the practice of directly quoting content. The implications of these results for ethical conduct in Twitter-based research are discussed next.

## Discussion

The results provide a first look at the predictors of Twitter users’ concern over being included in research in three settings and their expectations regarding consent and anonymity in publishing. While the survey showed a general lack of concern from users over their posts being used for research purposes (with university research attracting least concern), 80 per cent of respondents expected to be asked for their consent ahead of their Twitter content being published, and over 90 per cent stated they expected anonymity in publication (in particular female and BME tweeters and those posting personal photographs). These patterns reflect those found in the Eurobarometer Attitudes on Data Protection Survey (2011) that showed three-quarters of Europeans accepted that disclosing personal information was now a part of modern life, but only a quarter of respondents felt that they had complete control over their social media information and 70 per cent were concerned that their personal data may be used for a purpose other than for which they were archived. A clear majority of Europeans (75%) want to delete personal information on a website whenever they decide to do so, supporting the ‘right to be forgotten’ principle.

Taken together, these findings show that there may be a disjuncture between the current practices of social researchers in relation to publishing the content of Twitter posts, and users’ views of the fair use of their online communications in publications and their rights as research subjects. Much of this disconnection seems to stem from what is perceived as public in online communications, and therefore what can be published as data without consent or protection from anonymisation. Existing ethical guidelines that provide principles for research in public places focus on traditional forms of data and data collection. Most guidelines (e.g. BPS, BSA, International Visual Sociology Association) stress that consent, confidentiality and anonymity are often not required where the research is conducted in a *public place where people would reasonably expect to be observed by strangers*. However, the perceptions of the majority of users of Twitter clearly differ with this viewpoint. This is most likely because Twitter blurs the boundary between public and private space. However, it would be misleading to suggest this blurring is something new to researchers of public places:

What is public and what is private is rarely clear-cut. Is the talk among people in a public bar public or private? Does it make any difference if it is loud or *sotto voce*? Similarly, are religious ceremonies public events? It is not easy to answer these questions, and in part the answer depends on one’s point of view. (Hammersley and Atkinson, 1995: 267)

A social media researcher’s point of view must take to account the unique nature of this online public environment. Internet interactions are shaped by ephemerality, anonymity, a reduction in social cues and time–space distanciation (Joinson, 1998; Lash, 2001; Williams, 2006). Research has highlighted the disinhibiting effect of computer-mediated communication, meaning Internet users, while acknowledging the environment as a (semi-)public space, often use it to engage in what would be considered private talk. Marwick and boyd (2011) show how Twitter folds multiple audiences into a flattened context. This ‘context collapse’ creates tensions when behaviours and utterances intended for an imagined limited audience are exposed to whole actual audiences. Online information is often intended only for a specific (imagined) public made up of peers, a support network or specific community, not necessarily the Internet public at large, and certainly not for publics beyond the Internet (boyd, 2014). When it is presented to unintended audiences it has the potential to cause harm, as the information is flowing out of the context it was intended for (Barocas and Nissenbaum, 2014; Nissenbaum, 2009). In the final analysis, we may be satisfied with the AoIR (2012: 7) guidelines that state social, academic and regulatory delineations of the public–private divide may not hold in online contexts and as such ‘privacy is a concept that must include a consideration of *expectations* and *consensus*’ of users within context (emphases added).

Informed consent and anonymity are further warranted given the abundance of sensitive data that are generated and contained within these online networks. The models showed associations between sexual orientation, ethnicity and gender and feelings of concern and expectations of anonymity. A principal ethical consideration in most learned society guidelines for social research is to ensure the maximum benefit from findings while minimising the risk of actual or potential harm during data collection, analysis and publication. Potential for harm in social media research increases when sensitive data are estimated and published along with the content of identifiable communications without consent. These data can include sensitive personal demographic information (i.e. ethnicity and sexual orientation), information on associations (such as memberships to particular groups or links to other individuals known to belong to such groups) and communications of an overly personal or harmful nature (such as details on morally ambiguous or illegal activity and expressions of extreme opinion). In some cases, such information is knowingly placed online, whether or not the user is fully aware of who has access to this information and how it might be repurposed (Dicks et al., 2006). In other cases, sensitive information is not knowingly created by users, but it can often come to light in analysis where associations are identified between users and personal characteristics are estimated by algorithms (Van Dijck, 2013). Many recent Twitter-based research projects have reported encountering all three forms of sensitive information and several have used algorithms to generate classifications of users, including studies on demographic characteristics (Sloan et al., 2013, 2015) on the spread of cyberhate following terrorist events (Burnap and Williams, 2015, 2016; Burnap et al., 2014; Williams and Burnap, 2016), on racial tension (Burnap et al., 2013, 2014), on estimating offline crime patterns using online signals (Williams et al., 2017) and on suicidal ideation (Burnap et al., 2015a; Scourfield et al., 2016). In the latter case, algorithms were used to estimate the degree of emotional distress exhibited in user posts. If published alongside identifiable posts without consent, these classifications may stigmatise users and potentially cause further harm.

Taking the example of cyberhate on social media, Williams and Burnap (2016) employed machine learning algorithms to classify hateful content (see also Burnap and Williams, 2015). They report that automated text classification algorithms perform well on social media datasets around specific events. However, their accuracy decreases beyond the events around which they were developed due to changes in language use. Therefore, an ethical challenge arises about how researchers should develop, use and reuse algorithms that have the consequence of classifying content and users with sensitive labels often without their knowledge. Where text classification techniques are necessitated by the scale and speed of the data (e.g. classification can be performed as the data are collected in real-time), researchers should ensure the algorithm performs well (i.e. minimising the number of false positives) for the event under study in terms of established text classification standards. Furthermore, researchers have a responsibility to ensure the continuing effectiveness of the classification algorithm if there is an intention to use it beyond the event that led to its design. High-profile failures of big data, such as the inability to predict the US housing bubble in 2008 and the spread of influenza across the United States using Google search terms, have resulted in the questioning of the power and longevity of algorithms (Lazer et al., 2014). Algorithms therefore need to be openly published and transparent for reproducibility, such that they can be routinely tested for effectiveness, avoiding the mislabelling of content and users. Where classifications are published together with the qualitative content of communications, informed consent from quoted users must be sought to ensure they are in agreement with the labelling of their posts and happy for the content to be exposed to new audiences.

If we are to balance the privacy of Twitter users (the disinhibiting nature of the environment, context collapse and the abundance of sensitive information accepted) with the needs of research, a sensible way forward would be to continue to programmatically collect data without explicit consent, but seek informed consent for all directly quoted content in publications. Providing total anonymity to directly quoted users is not practical in this form of research, due to Twitter guidelines and the issue of online search (where quoted text is easily searchable rendering users and their partners in conversation identifiable). Therefore, users must be made aware of this limitation during consent negotiations. In the case of the reproduction of tweets (public display of tweets by any and all means of media) Twitter (2016) Broadcast guidelines state publishers should:

i) Include the user’s name and Twitter handle (@username) with each Tweet;
ii) Use the full text of the Tweet. Editing Tweet text is only permitted for technical or medium limitations (e.g., removing hyperlinks);
iii) Not delete, obscure, or alter the identification of the user. Tweets can be shown in anonymous form in exceptional cases such as concerns over user privacy;
iv) In some cases, seek permission from the content creator, as Twitter users retain rights to the content they post.

Informed consent is further warranted considering Twitter’s view that users retain rights to the content they post and that they have a ‘right to be forgotten’. Twitter (2015) terms of service for the use of their APIs by developers require that data harvesters honour any future changes to user content, including deletion. As academic papers cannot be edited continuously post-publication, this condition further complicates direct quotation without consent (needless to mention the burden of checking content changes on a regular basis). However, researchers should not conclude that conventional representation of qualitative data in social media research is rendered too burdensome. As in conventional qualitative research, researchers can make efforts to gain informed consent from a limited number of posters if verbatim examples of text are required. Where the content to be quoted is not sensitive and the user is not vulnerable (see below), researchers may be satisfied with *opt-out* consent. In cases where consent is not provided to direct quote without anonymisation, Markham (2012) advocates a bricolage-style reconfiguration of original data that represents the intended meaning of interactions. This can include creating composite accounts and posts by selecting representative elements from the data and composing a new original that is not traceable back to an identifiable individual or interaction. Such a reconstruction is accomplished via close attention to context, to avoid the loss or change of meaning. However, this reconfiguration must be extensive enough not to contravene Twitter guidelines – effectively meaning data are fictionalised for presentation purposes. While this may be suitable for general thematic analysis, it may not satisfy the needs of more fine-grained approaches, such as those undertaken by interactionist scholars.

**Figure 1.**
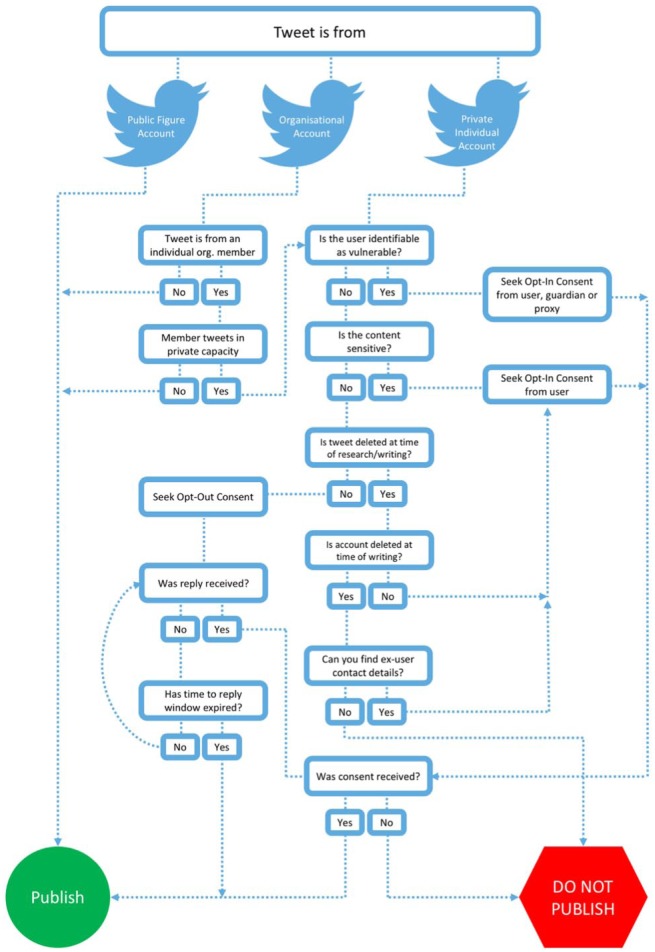
Decision flow chart for publication of Twitter communications.

In line with the points raised in this article we propose the following framework for researchers and research ethics committees considering research using Twitter data (that can be extrapolated to other forms of public social media data):

- Conduct risk assessment in relation to publishing verbatim Twitter content posted by users. Figure 1 presents a flow chart that can be consulted to reach decisions on the publication of Twitter communications. In seeking to reach decisions on the status of tweeters (e.g. public, organisational, private, vulnerable) and their tweets (e.g. organisational, private, sensitive) researchers should consult existing ethical guidelines that provide definitions of public figures (e.g. politicians and celebrities who aim to communicate to a wide audience), vulnerable individuals (e.g. children, learning disabled and those suffering from an illness) and sensitive content (e.g. posts about criminal activity, financial problems, mental health issues and feelings of suicide, extramarital sexual activity, controversial political opinions and activism) (Townsend and Wallace, 2016). As social media accounts can lack personal details, and it is difficult to find additional identifying details, researchers may be satisfied with the use of the information presented in the profile and in posts alone to reach decisions on the status of users. In the case of opt-out consent, researchers may wish to set a reasonable time window for a reply (e.g. two weeks to one month), and repeat consent requests several times should a timely response not be forthcoming. If the tweeter is no longer active (i.e. has not tweeted in last three to six months) consider the account as inactive and do not publish (as we can reasonably assume the tweeter has not seen the request and therefore cannot take up the option of opting-out).
- Photos and videos (excluding general non-personal images), that are included in a tweet should be considered as sensitive information. Opt-in informed consent should be sought before media content is published.
- Ensure location data (especially latitude and longitude coordinates obtained from GPS) within Twitter posts are published only with explicit opt-in consent from the user, and that location data are stored with the original tweet in any dataset. A user should consent to the way in which their location is to be displayed in the publication.
- If using software that enhances Twitter data, ensure algorithms that derive characteristics and classify users are open to researchers and research subjects. The accuracy, configurations and threshold settings of algorithms should be made public to ensure reproducible research. If sensitive personal classifications (e.g. ethnicity and sexual orientation) are to be published with tweet content, seek opt-in consent from users.
- Twitter data can be reused by others beyond the researcher or team who collected it up to a limit of 50,000 tweets per day (if shared manually, such as via a .csv (spreadsheet) file delivered via email or on a USB memory stick). Larger datasets can be distributed in short time periods by sharing the Tweet IDs and/or User IDs only (numerical identifiers unique to individual tweets/users). These IDs can be used to revivify the whole dataset via a new query to the Twitter API. This process protects the privacy of users who have deleted or altered content beyond the original point of data collection. The UK Data Archive currently allows for the storing of Tweet IDs for data re-use.

## Conclusion

Researchers are now able to freely harvest social data at a hitherto unrealised scale and speed through public social media APIs. Codes of ethical conduct that were first written over half a century ago are being relied upon to guide the collection, analysis and representation of digital data. The result has been a rush to have a go without the benefit of the full picture. In some cases, researchers have simply considered online communications as public data, representing them in publication verbatim without consent or anonymisation. These researchers may have benefitted from knowing more about the views of social media users, an appreciation of the effect of context collapse and online disinhibition on the behaviours of users, and a fuller understanding of how algorithms classify users with potentially sensitive labels. However, there are mitigating factors, as social media companies’ terms of service for the use of their APIs promote the practice of data harvesting, and in some cases forbid the anonymisation of content in all forms of reproduction. However, these terms of service were not written with social researchers in mind. Researchers need to interpret and engage with terms of service through the lens of social science research that implies a more reflexive ethical approach than provided in legal accounts of the permissible use of these new forms of data. We hope that this article acts as a clarion call for additional research in this area. Future research might consider: (1) the ethical issues associated with the repurposing of Twitter, and other social media data, in ways that may have undesirable consequences beyond privacy issues that users may object to, such as the use of posts to develop evidence and arguments for a particular political position that is at odds with users’ affiliations; (2) the functioning of text classification algorithms to estimate personal characteristics, and their use in various research settings; (3) the ethical issues raised by the non-consensual identifiable publication of different types of social media content, such as images, videos and URLs; and (4) the views of social media companies towards the use of data generated on their networks for research purposes.

## Notes

1. The methods sections in these articles do not make it clear if attempts were made to gain informed consent from Twitter users to publish original content. The content published is searchable (e.g. via Twitter Advanced Search and Google) and therefore traceable back to individual user accounts and associated personal information (such as profile descriptions and location data). Where a search returns no result the content and/or account has been deleted by the user or Twitter.

2. One petabyte is equivalent ~1 billion megabytes.

3. The BSA launched its updated guidance following the 2017 Annual Conference. The BSA Digital Sociology Study Group drafted the new guidelines for research in online settings. The new BSA Digital Research Annex is available online at: www.britsoc.co.uk/media/24309/bsa_statement_of_ethical_practice_annexe.pdf ; and the case studies are available online here: http://digitalsoc.wpengine.com/?cat=126.

4. www.legislation.gov.uk/ukpga/1998/29/schedule/3.

5. We do not intend to provide a one-sided view of the use of algorithms in this article, and acknowledge the importance of sharing algorithmic estimations precisely so certain groups can be protected in research (e.g. classifying age so that researchers can exclude children from analysis and publication).

6. See www.survey.bris.ac.uk/.

7. A nonparametric resampling procedure was used to empirically estimate the sampling distribution of the indirect effect, thus reducing problems with type I errors and low statistical power endemic to analyses that rely on assumptions of sampling distribution normality.

8. These distinctions were derived from discussions with our Lab advisory panel made up of government, commercial and public social media data users.
